# Construction Notes

**Published:** 1893-03-25

**Authors:** 


### CONSTRUCTION NOTES.

The Committee of the Leek Cottage Hospital in referring, at their annual meeting, to a piece of land at the rear of the hospital, the gift of their chairman, report that, conjointly with the trustees of the institution, they have purchased a piece of land which will protect the hospital from outside encroachment, and be of great service to the inmates, at the cost of £150, and subscriptions amounting to £74 have already been received. New oaken gates have been provided by Mrs. J. Alsop, which will shortly be placed in position, and Mrs. Alsop has erected a memorial archway at the entrance of the hospital in memory of her husband.

After much earnest consideration the Committee of Management of the Dental Hospital of London have decided to purchase a new site, and to build a new hospital, it having been thought "that reconstruction upon the present site is out of the question, and that the true interests of both hospital and school will be best served by a new building on a new site of sufficient area, with ample possibilities of lighting." This conclusion was forced upon the Committee for the following reasons: The waiting rooms are uncomfortable, unhealthy, and the men's department without sanitary accommodation. The operating rooms are used as passages, and the entrance to the anæsthetic room dangerous. The dental appliance departments are inadequate both for patients and students. Although possessing an admirable façade, many parts of the building are only twelve feet deep. The hospital having been constructed by the alteration of old houses, the whole of the sanitary arrangements are unsatisfactory. Hot and cold water is not fitted to any room in the hospital. The building is imperfectly heated. The students have not proper accommodation in cloak room, lavatories, or sitting-room. £40,000 will be required to carry out the proposed change, and of this sum £6,000 has already been promised by the staff, the past and present students of the hospital, and some members of the dental profession, an additional £2,000 having been subscribed by old friends of the hospital. The Duke of Cambridge, the President, cordially supports the idea of the proposed new hospital, and the Prince of Wales has sent a donation towards the amount required.

